# Barriers to effective diabetes management – a survey of people with severe mental illness

**DOI:** 10.1186/s12888-018-1744-5

**Published:** 2018-06-01

**Authors:** Kathleen Mulligan, Hayley McBain, Frederique Lamontagne-Godwin, Jacqui Chapman, Chris Flood, Mark Haddad, Julia Jones, Alan Simpson

**Affiliations:** 1 School of Health Sciences, City, University of London, Myddelton Street, London, EC1V 0HB UK; 2 East London NHS Foundation Trust, London, UK; 3 School of Human and Social Sciences, University of West London, London, UK; 4 Diabetes Specialist Nursing Service, East London NHS Foundation Trust, London, UK; 5 Centre for Research in Primary & Community Care (CRIPACC), University of Hertfordshire, Hatfield, Hertfordshire UK

**Keywords:** Diabetes, Severe mental illness, Service users, Self-management, Theoretical domains framework

## Abstract

**Background:**

People with severe mental illnesses (SMI) such as schizophrenia and bipolar disorder have an increased risk of developing type 2 diabetes and have poorer health outcomes than those with diabetes alone. To maintain good diabetes control, people with diabetes are advised to engage in several self-management behaviours. The aim of this study was to identify barriers or enablers of diabetes self-management experienced by people with SMI.

**Methods:**

Adults with type 2 diabetes and SMI were recruited through UK National Health Service organisations and mental health and diabetes charities. Participants completed an anonymous survey consisting of: Summary of Diabetes Self-Care Activities (SDSCA); CORE-10 measure of psychological distress; a measure of barriers and enablers of diabetes self-management based on the Theoretical Domains Framework; Diabetes UK care survey on receipt of 14 essential aspects of diabetes healthcare. To identify the strongest explanatory variables of SDSCA outcomes, significant variables (*p* < .05) identified from univariate analyses were entered into multiple regressions.

**Results:**

Most of the 77 participants had bipolar disorder (42%) or schizophrenia (36%). They received a mean of 7.6 (SD 3.0) diabetes healthcare essentials. Only 28.6% had developed a diabetes care plan with their health professional and only 40% reported receiving specialist psychological support. Engagement in self-management activities was variable. Participants reported taking medication on 6.1 (SD 2.0) days in the previous week but other behaviours were less frequent: general diet 4.1 (2.3) days; specific diet 3.6 (1.8) days, taking exercise 2.4 (2.1) days and checking feet on 1.7 (1.8) days. Smoking prevalence was 44%. Participants reported finding regular exercise and following a healthy diet particularly difficult. Factors associated with diabetes self-management included: the level of diabetes healthcare and support received; emotional wellbeing; priority given to diabetes; perceived ability to manage diabetes or establish a routine to do so; and perceived consequences of diabetes self-management.

**Conclusions:**

Several aspects of diabetes healthcare and self-management are suboptimal in people with SMI. There is a need to improve diabetes self-management support for this population by integrating diabetes action plans into care planning and providing adequate psychological support to help people with SMI manage their diabetes.

**Electronic supplementary material:**

The online version of this article (10.1186/s12888-018-1744-5) contains supplementary material, which is available to authorized users.

## Background

People with severe mental illnesses (SMI), such as schizophrenia and bipolar disorder, have poorer physical health than the general population [1, 2]. Among the health disparities experienced is a two-fold risk of developing diabetes [3, 4], which has a prevalence of approximately 13% in people with SMI [5]. Diabetes outcomes are also poorer in this group as they experience a higher risk of acute [6] and macrovascular complications [7] and higher mortality [8] than those with diabetes alone.

To maintain good diabetes control and thus reduce the risk of complications, people with diabetes are advised to engage in several self-management behaviours. These may include taking medication, eating a healthy diet, taking regular physical activity, giving up smoking, monitoring blood glucose levels, examining their feet and attending regular health checks, including retinopathy screening. The DAWN2 cross-national survey of diabetes concluded that performance of these behaviours in the general diabetes population is sub-optimal in most countries, particularly for glucose monitoring, physical activity and foot care [9]. A study in Taiwan that used the same measure of self-management behaviour as DAWN2 found that performance was poorer in people with diabetes and schizophrenia than in those with diabetes alone [10].

Very little research has explored factors that may influence diabetes self-management in people with SMI. In a recent qualitative interview study that informed the current research [11], we found that suboptimal diabetes self-management in people with SMI did not appear to be explained by a lack of knowledge of the recommended self-management behaviours or of the potential consequences of poor diabetes control; people with SMI reported awareness of both but found it difficult to adopt and/or maintain the recommended behaviours. Barriers to effective diabetes self-management that have been found among people with SMI include psychological factors such as stress and isolation [12] or periods of deteriorating mental health [11]; low self-efficacy [10], lack of social support [12] and poor relationships with health providers or fragmented care [12]. Conversely, receipt of support from family and health professionals has been reported as an enabler of diabetes self-management [11].

The aim of the current study was to enhance our understanding of factors that may influence diabetes self-management among people with SMI by using a theoretical framework to explore a comprehensive range of potential barriers and enablers. The findings will inform the development of an intervention to help support diabetes self-management among people with SMI.

## Methods

### Design

An anonymous cross-sectional survey was conducted between November 2015 and October 2016.

### Participants

People were eligible to participate if they:

- were aged 18 years or over,
- had a SMI (defined as schizophrenia, schizoaffective disorder, bipolar disorder or depression with psychotic features)
- had type 2 diabetes
- were able to read English

In the case of participants who were recruited through the UK National Health Service (NHS) (see below), a member of the clinical team checked eligibility criteria, including diagnoses, before sending out the survey, however, this check was not possible in the case of participants recruited through other routes.

### Procedures

Following advice received from patient and public involvement (PPI) representatives, recruitment was undertaken through both NHS organisations, relevant charities and service user networks. These included a national diabetes charity, a community database of people with diabetes in South West England, five national and five local mental health charities (four in London and one in South West England), one national service user network and a local service user group in London. The NHS organisations were five NHS Trusts (three in North and East London, one in South West England and one in East England) and 16 general practice (GP) surgeries (15 in North and East London and one in a city in the Midlands) .

The charities and service user groups advertised the survey through their websites and/or newsletters and provided a link to the online version of the survey. NHS organisations identified eligible service users from their databases and posted a paper version of the survey questionnaire to them with a freepost envelope for return of the questionnaire to the research team. The correspondence also contained details of the link to the online version of the survey, which participants could complete instead of the paper version, if preferred. Participants were advised that a contribution of £2 would be made to a diabetes or mental health charity for each completed questionnaire.

### Measures

The survey was developed in collaboration with established research advisory groups of mental health service users and carers [13] and people with diabetes [14]. The survey comprised questions on the following:

- Performance of diabetes self-management behaviour was the primary study outcome and was assessed using the Summary of Diabetes Self-Care Activities (SDSCA) scale [15]. This is a validated and widely used measure of performance of diabetes self-management. The measure has 11 core items - self-monitoring of blood glucose (2 items), foot care (2 items), general diet (2 items), specific diet items about eating fruit and vegetables and high fat foods (2 items), physical activity (2 items) and smoking status (1 item); we also included two of the optional items about medication (2 items). Participants are asked on how many of the last 7 days they performed each behaviour. Summary scores are calculated for each behaviour using the mean number of days. For smoking, participants are asked whether or not they smoked in the last 7 days, producing a categorical outcome variable. We added a question asking participants to indicate which one of these aspects of their diabetes they find most difficult to manage.
- Psychological distress over the previous week was assessed using the CORE-10 [16]. This validated measure comprises 10 items scored on a 5-point scale ranging from 0 (‘not at all’) to 4 (‘most or all the time’). A total score is calculated by adding the values of all 10 items to give a score from 0 to 40, with a higher score representing greater psychological distress. A score of ≤10 is in the non-clinical range, a score of 11–14 is considered ‘mild’ distress, 15–19 ‘moderate’, 20–24 ‘moderate-to-severe’ and 25+ ‘severe’ psychological distress.
- Barriers and enablers of diabetes self-management were examined using a 27-item questionnaire. Questionnaire items were generated from a previous interview study with service users about the barriers and enablers to diabetes self-management they experience [11]. The interview schedule and subsequent questionnaire were based on the Theoretical Domains Framework (TDF) [17], a synthesis of 33 theories of behaviour change, which comprises 14 theoretical domains found to influence behaviour. The 14 domains are 1) knowledge, 2) skills, 3) social/professional role and identity, 4) beliefs about capabilities, 5) optimism, 6) beliefs about consequences, 7) reinforcement, 8) intentions, 9) goals, 10) memory, attention and decision processes, 11) environmental context and resources, 12) social influences, 13) emotion and 14) behavioural regulation. The questionnaire included items which were deemed relevant from the interview study, and covered each of these 14 domains (see Additional file 1). Items were scored on a 7-point Likert scale ranging from ‘strongly disagree’ to ‘strongly agree’, with higher sores representing stronger agreement. Subscales representing the 14 domains of the barriers and enablers questionnaire were created by calculating means of the items in each domain. Items 6 and 18 (see Additional file 1) were reverse-scored.
- The Diabetes UK care survey was used to measure participants’ experience of receiving recommended diabetes care [18]. The survey explores whether respondents have received diabetes ‘healthcare essentials’ [19] to which the response options are ‘Yes’, ‘No’ or ‘Don’t Know’. We excluded three questions that ask about care for children with diabetes, giving a 15-item measure, 14 of which ask about receipt of healthcare essentials plus one that asks if the quality of diabetes care received over the past 12 months had improved, stayed the same or worsened. The total number of healthcare essentials received was calculated. In addition, we asked participants to indicate if they would be interested in receiving diabetes education (if they had not already received any) or more education (if they had already attended diabetes education).
- Demographic characteristics (age, education, employment status, ethnicity, gender, relationship status).
- Clinical characteristics: participants were given a list of mental health diagnoses and asked to tick all that applied to them. They were also asked how long ago, in years, they were diagnosed with a) diabetes and b) their mental health problem. Participants were asked how they managed their diabetes (response options: tablets; insulin; lifestyle; don’t know) and which medication they take for their mental health (free text response box).

### Statistical analysis

Data were analysed using IBM SPSS Statistics v.23.

Composite variables representing the 14 domains of the barriers and enablers questionnaire were created. To test the internal consistency, inter-item correlations were calculated for each domain. In the case of scales with fewer than 10 items it is more appropriate to report the mean inter-item correlation, rather than Cronbach alpha. It is recommended that average inter-item correlations should fall between 0.15 and 0.50 [20]. Inter-item correlations were found to range from 0.31 to 0.73, with two domains (*Memory, Attention and Decision Processes*; *Environmental Context and Resources*) exceeding the recommended higher limit of 0.50. The composite variables were used in the remainder of the analyses.

To examine which factors were associated with performance of self-management behaviours, univariate analyses were initially performed. The independent variables (IVs) were demographic and clinical variables, psychological distress, diabetes care received and the 14 domains from the barriers and enablers questionnaire. The relationship between SDSCA variables and continuous IVs was examined by Pearson r correlations. In the case of categorical IVs (e.g. gender), differences in SDSCA between categories were examined by t-test or analysis of variance (ANOVA), as applicable.

To examine which were the strongest explanatory variables of SDSCA outcomes, significant IVs (*p* < .05) identified from the univariate analyses were included in stepwise multiple linear regressions. Separate regression analyses were performed for each of the behaviours assessed in the SDSCA. As smoking was a categorical variable, logistic regression analysis was performed for this outcome. Before entering categorical IVs into the regressions, dummy variables were created for those with more than two categories.

To enter all of the 26 IVs outlined above in the multiple regression analysis, based on a medium effect size of 0.15, with 80% power and alpha 0.05, would require a sample size of 175. However, only variables that were significantly associated with the outcome of interest in the univariate analysis (*p* < 0.05) were entered into the multiple regression analyses.

## Results

A total of 486 questionnaires were mailed out to service users. Ninety-seven people opened the online survey, of whom 70 consented into the study, and 52 people completed the paper survey, giving a total sample of 122 participants. Eleven participants were excluded as they reported mental health and/or diabetes diagnoses that did not meet the study inclusion criteria. We excluded cases who had missing data for more than 50% of variables, a threshold above which imputation is not recommended [21, 22]. This gave a final sample of 77 (63.1%). Little’s Missing Completely at Random (MCAR) test was non-significant (Chi-squared = 443.641, df = 444, *p* = 0.496), indicating that data were MCAR. Missing data were managed using multiple imputation methods in IBM SPSS version 23. Full Conditional Specification (FCS), an interactive Markov chain Monte Carlo (MCMC) procedure, was used as the missing data pattern was arbitrary. Constraints and rounding were used to ensure that the imputed scale level data was meaningful and corresponded to possible values. The model used to generate the imputed values corresponded with the model used for the analysis. Ten scale level imputation iterations were used to eliminate bias; it has been suggested that between 3 and 10 imputations are sufficient [23]. All analysis was performed on each of these 10 datasets and then pooled to give a final result.

Participant demographic and clinical characteristics are shown in Table 1. A majority of participants were male, of white British ethnicity, with an average age of 52.3 years (SD 11.5). Just over half of the sample were educated to A-Level standard (a national exam taken at 18 years of age) or above but this varied from 70.3% of those with bipolar disorder, 58.8% of those with depression with psychotic features, 50% with schizoaffective disorder and 44.4% of participants with schizophrenia. Few participants were in employment. Approximately half of the sample was living alone. Participants had been diagnosed with SMI for an average of 19.5 years and with diabetes for an average of 5 years. Most participants were taking tablets to manage their diabetes, but 10 (13%) were taking insulin. The most commonly occurring SMI in our sample was bipolar disorder, followed by schizophrenia. Over half of the sample reported experiencing at least moderate psychological distress over the previous week (Table 2).

**Table 1:** Demographic and clinical characteristics

Age in years, mean (SD)	52.3 (11.5)
Gender, n (%) Male	41 (53.2)
Ethnicity, n (%)	
White, British	47 (61.0)
White, other	7 (9.1)
South Asian	8 (10.4)
Black African Caribbean	6 (7.8)
Other	8 (10.4)
Missing data	1 (1.3)
Relationship status, n (%)	
Married/Living with Partner	20 (26.0)
Living alone	39 (50.6)
Living with relatives/friends/supported accommodation	16 (20.8)
Missing data	2 (2.6)
Education – highest qualification, n (%)^a^	
Higher education/professional or vocational equivalent	27 (35.1)
A Levels / vocational level 3 or equivalent	13 (16.9)
GCSE A^*^- C/O Level/vocational level 2 or equivalent	13 (16.9)
None or Qualifications at level 1 and below	13 (16.9)
Other qualifications: level unknown	8 (10.4)
Missing data	3 (3.9)
Employment	
Full time work	4 (5.2)
Part time work	3 (3.9)
Full time homemaker	3 (3.9)
Unemployed	33 (42.9)
Other	8 (10.4)
Retired	7 (9.1)
Missing	19 (24.7)
Diabetes duration (years), median (IQR)	5.0 (2.0–9.0)
Diabetes medication, n, %	
Tablets only	52 (67.5)
Insulin only	4 (5.2)
Tablets and insulin	6 (7.8)
Lifestyle only	15 (19.5)
SMI duration (years), median (IQR)	19.5 (10.75–30.25)
SMI diagnosis, n, % (may have more than one diagnosis)	
Schizophrenia	28 (36.4)
Schizoaffective disorder	13 (16.9)
Depression with psychotic features	17 (22.1)
Bipolar disorder	32 (41.6)

^a^A levels – national exams taken at age 18 years; GCSE – national exams taken at age 16 years

**Table 2:** Mental Health

CORE-10 measure of global distress, mean (SD)	16.65 (7.81)
CORE-10 classifications, n, %	
Non-clinical range 0–10	18 (23.4)
Mild 11–14	16 (20.8)
Moderate 15–19	16 (20.8)
Moderate to severe 20–24	14 (18.2)
Severe 25+	13 (16.9)

Scale 0–40, higher score = more distress. A score of ≤10 = non-clinical range, score 11+ = clinical range – 11-14 = mild, 15–19 = moderate, 20–24 = moderate-to-severe, 25+ = severe

### Diabetes self-management behaviour

The average number of days on which participants reported engaging in recommended self-management behaviours is shown in Table 3. Following recommended behaviour was most common for medication and least common for exercise and foot care. Participants reported eating a healthy diet roughly half of the time. The aspects of diabetes self-management that participants reported finding most difficult were taking regular exercise and eating a healthy diet. Over 40% of participants had smoked in the past 7 days and of these, only 21% considered not smoking to be the most difficult aspect of diabetes management.

**Table 3:** Diabetes self-management behaviour

Summary of Diabetes Self-Care Activities (SDSCA)	Mean	SD
Number of days in last 7 that participant has engaged in		
Taking medication	6.1	2.0
Blood sugar testing^a^	3.8	2.7
General diet	4.1	2.3
Specific diet	3.6	1.8
Exercise	2.4	2.1
Foot Care	1.7	1.8
	*n*	%
Smoked	34	44.2
Which aspect of your diabetes do you find most difficult to manage?		
Exercising for at least 30 min, 5 days a week	29	37.7
Following a healthy eating plan	27	35.1
Not smoking	7	20.6^b^
Testing blood sugar	5	6.5
Taking diabetes medication	5	6.5
Checking feet	4	5.2

^a^not all participants had been supplied with a blood glucose monitor – these data relate to 40 participants who had received a monitor

^b^of those who reported being smokers

### Diabetes care received

Table 4 shows the number of participants who reported receiving each of the diabetes healthcare essentials. The responses of participants in the Diabetes UK survey [18] are also shown for comparison. Participants in the current study reported receiving a mean of 7.6 (SD 3.0) of the 14 healthcare essentials. Almost a quarter of the sample reported that their diabetes care had improved over the past 12 months, but for 14% it had worsened. The most commonly received aspects of care were blood pressure and eye checks, received by 85.7 and 83.1% of participants respectively. The least common were: being seen by a diabetes specialist if in hospital (20.3%); developing a care plan with their healthcare professional (28.6%); and being offered specialist psychological support (40.3%). Other aspects of care were each received by approximately two-thirds of participants.

**Table 4:** Diabetes healthcare essentials received

	Current study		Diabetes UK survey	
	n	%	n	%
Over the past 12 months, in general has the overall quality of diabetes care that you receive:				
Improved	18	23.4	709	10.6
Stayed the same	41	53.2	4776	71.3
Worsened	11	14.3	851	12.7
I don’t know	7	9.1	366	5.5
In the last year, or as part of your most recent annual review, have you had an HbA_1c_ blood test carried out, to measure your overall blood glucose control?				
Yes	56	72.7	6319	94.2
No	7	9.1	389	5.8
Don’t know	14	18.2		
In the last year, or as part of your most recent annual review, have you had your blood pressure measured?				
Yes	66	85.7	6225	92.8
No	5	6.5	480	7.2
Don’t know	6	7.8		
In the last year, or as part of your most recent annual review, have you had your blood fats (cholesterol) measured?				
Yes	53	68.8	5730	85.8
No	12	15.6	945	14.2
Don’t know	12	15.6		
Your eyes should be screened for retinopathy each year, using a specialised digital camera. In the last year, or as part of your most recent annual review, have you had your eyes looked at?				
Yes	64	83.1	6271	93.4
No	13	16.9	446	6.6
In the last year or as part of your most recent annual review, have you had your legs and feet checked?				
Yes	50	64.9	5131	76.4
No	25	32.5	1587	23.6
Don’t know	2	2.6		
In the past year, or as part of your most recent annual review, have you had a urine or blood test to monitor your kidney function?				
Yes	50	64.9	5163	78.6
No	19	24.7	791	12.0
Don’t know	8	10.4	616	9.4
In the last year, or as part of your most recent annual review, have you had your weight checked and your waist measured?				
Yes	52	67.5	4549	69.5
No	22	28.6	2000	30.5
Don’t know	3	3.9		
If you are a smoker, in the last year, or as part of your most recent annual review, have you had support and advice on how to quit?				
Yes	23	62.2	366	56.5
No	11	29.7	282	43.5
Don’t know	3	8.1		
Don’t smoke	40		5904	
Have you developed a care plan with your healthcare professional, which has been decided by discussing your individual needs with them to set targets?				
Yes	22	28.6	2339	35.9
No	55	71.4	4184	64.1
Have you ever been offered an education course about diabetes, either when you were first diagnosed or later on?				
Yes	50	64.9	3676	56.2
No	27	35.1	2867	43.8
Don’t know			65	13.7
If you have had to go into hospital, for whatever reason, has somebody from a diabetes specialist team come to see you about your diabetes while you were there?				
Yes	14	20.3	803	30.4
No	55	79.7	1835	69.6
Not applicable	8		3667	
If you are planning a pregnancy, or you are pregnant, have you been offered specialist healthcare advice before and during your pregnancy?				
Yes	4	40.0	158	60.1
No	6	60.0	105	39.9
Not applicable	67		3667	
Do you think that, when the need arises, you are referred to the care of specialists who can help you with different aspects of your diabetes?				
Yes	53	68.8	4294	69.4
No	24	31.2	1894	30.6
If you have needed it, have you been offered emotional or psychological support from a specialist healthcare professional or service?				
Yes	31	40.3	723	24.1
No	46	59.7	2277	75.9
Not applicable			3293	

Of the 50 participants who had been offered diabetes education, 40 (80.0%) reported that they would be interested in receiving more education. Of 27 participants who had not been offered education, 19 (70.4%) reported that they would be interested in receiving education.

### Barriers and enablers to performing diabetes self-management behaviours

**Fig. 1:**
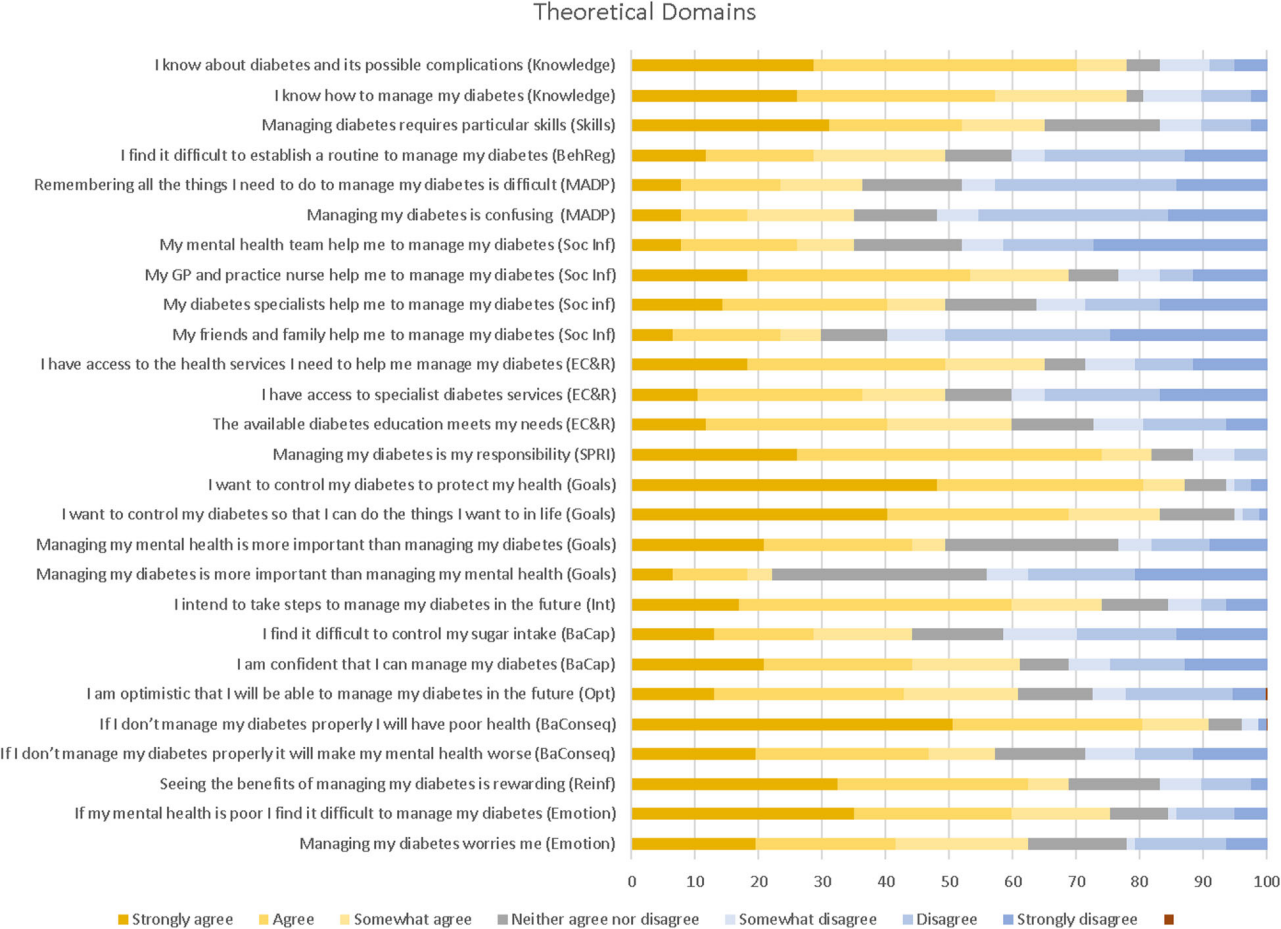
Responses to questions in the Theoretical Domains Framework questionnaire

Responses concerning the individual barriers and enablers are shown in Fig. 1. A majority of respondents (> 75%) agreed that they know about diabetes and how to manage it. They were also aware that poor diabetes control would have adverse health consequences, with almost all participants reporting that if they did not manage their diabetes properly they would have poor health. However, a smaller percentage (57.2%) felt that poor diabetes management would have an impact on their mental health.

Most people saw diabetes management as their responsibility and intended to take steps to manage their diabetes in the future. Diabetes management was considered important, with over 80% wanting to control their diabetes to protect their health and do the things they want to in life; however managing mental health was considered more important than managing diabetes by approximately half the sample.

Just over 60% felt confident in their ability to manage their diabetes and felt optimistic that they would be able to do so in future. However, difficulty in establishing a routine to manage diabetes was reported by approximately half of the respondents and about a third reported that they found managing diabetes confusing or struggled to remember all the things they needed to do to manage their diabetes. Controlling sugar intake was reported as difficult by over 40% of participants. Managing diabetes was a worry for over 60% of participants and 75% reported that they find it difficult to manage their diabetes if their mental health is poor.

The extent to which other people were perceived as helping with diabetes management varied, with over two-thirds (69%) agreeing that primary care staff helped them to manage their diabetes, but this was lower for diabetes specialists (49%) and their mental health team (35%) and only 30% felt friends and family helped them to manage their diabetes.

#### Factors associated with diabetes self-management

Univariate associations between diabetes self-management behaviours (SDSCA variables) and continuous and categorical IVs are shown in Tables 5 and 6 respectively. Several factors were significantly correlated (*p* < 0.05) with one or more self-management behaviour, but none were significant across all behaviours. Variables that were statistically significant in the multiple regression analyses and logistic regression analysis are shown in Tables 7 and 8 respectively. Findings for each of the self-management behaviours are summarised below.

**Table 5:** Univariate relationships between continuous independent variables and self-management behaviours

	Summary of Diabetes Self Care Activities variables						
	Pearson correlations (*p* value in brackets)						t-test (*p* value)
	Blood Glucose Testing	Medication	General Diet	Specific Diet	Exercise	Foot Care	Smoking
Age	.045	.047	.243	.120	.172	.139	1.154
	(.818)	(.766)	(.071)	(.381)	(.206)	(.310)	(.249)
Diabetes duration in years	−.140	−.204	−.138	−.023	.066	−.203	1.471
	(.439)	(.141)	(.265)	(.854)	(.598)	(.100)	(.141)
SMI duration in years	−.180	−.076	.169	.196	.177	.059	−.389
	(.295)	(.588)	(.162)	(.105)	(.144)	(.627)	(.697)
Diabetes healthcare essentials_total	−.144	.277	.371	.139	.264	.173	.129
	(.386)	(.030)	(.001)	(.231)	(.021)	(.136)	(.897)
CORE Total Score	.334	−.065	−.250	−.082	−.304	−.078	−.265
	(.037)	(.625)	(.028)	(.478)	(.007)	(.500)	(.791)
TDF Behavioural Regulation	−.344	−.236	−.348	−.344	−.216	−.159	1.188
	(.031)	(.067)	(.002)	(.002)	(.059)	(.168)	(.235)
TDF Beliefs about Capabilities	.091	.146	.466	.350	.313	.117	−1.228
	(.585)	(.259)	(.000)	(.002)	(.005)	(.315)	(.220)
TDF Beliefs about Consequences	.250	−.045	.297	.090	.175	−.088	1.507
	(.125)	(.734)	(.008)	(.437)	(.128)	(.446)	(.132)
TDF ECR	.088	.023	.382	.269	.333	.220	.602
	(.597)	(.860)	(.001)	(.018)	(.003)	(.055)	(.547)
TDF Emotion	−.115	−.136	−.371	−.180	−.210	−.263	2.631^a^
	(.486)	(.294)	(.001)	(.120)	(.067)	(.021)	(.009)
TDF Goals	−.076	.172	.547	.377	.328	.065	.628
	(.646)	(.184)	(.000)	(.001)	(.003)	(.574)	(.530)
TDF Intention	−.036	−.107	.301	.202	.236	.151	−.480
	(.830)	(.412)	(.008)	(.080)	(.042)	(.191)	(.631)
TDF Knowledge	.173	.040	.261	−.005	.129	.058	1.799
	(.296)	(.763)	(.022)	(.966)	(.263)	(.619)	(.072)
TDF MADP	−.100	.098	−.272	−.158	−.156	−.161	.217
	(.547)	(.459)	(.016)	(.174)	(.178)	(.165)	(.828)
TDF Optimism	−.142	.240	.414	.229	.092	.188	.534
	(.391)	(.062)	(.000)	(.045)	(.432)	(.103)	(.593)
TDF Reinforcement	−.059	.062	.475	.233	.232	.100	1.157
	(.723)	(.638)	(.000)	(.042)	(.042)	(.391)	(.247)
TDF Skills	.059	.138	.135	.138	.105	.148	−.502
	(.722)	(.294)	(.243)	(.233)	(.367)	(.200)	(.616)
TDF Social Influence	−.176	.157	.392	.315	.291	.256	0.330
	(.283)	(.226)	(.000)	(.005)	(.010)	(.025)	(.741)
TDF SPRI	−.108	−.240	−.136	−.128	−.074	.052	1.008
	(.524)	(.063)	(.240)	(.272)	(.523)	(.659)	(.313)

^a^Non-smokers more likely to agree with emotion items

ECR – Environmental Context and Resources; MADP – Memory, Attention and Decision Processes; SPRI – Social/Professional Role and Identity

**Table 6:** Univariate relationships between categorical independent variables and self-management behaviours

	Summary of Diabetes Self Care Activities variables												
	Blood glucose testing		Medication		General diet		Specific diet		Exercise		Foot care		Smoker/ non-smoker
	Mean	Statistic *P* value	Mean	Statistic *P* value	Mean	Statistic *P* value	Mean	Statistic *P* value	Mean	Statistic *P* value	Mean	Statistic *P* value	Statistic *P* value
Gender													
Male	4.2085	*t* = 0.757	6.42	*t* = 1.426	4.4878	*t* = 1.683	3.4504	*t* = −0.865	2.5976	0.949	1.6724	*t* = −0.024	**χ**^**2**^ **= 4.467**
Female	3.6591	*p* = 0.454	5.66	*p* = 0.159	3.5893	*p* = 0.097	3.8154	*p* = 0.390	2.1324	0.346	1.7453	*p* = 0.981	***p*** **= 0.039**
Ethnicity													
Asian	4.4000	F = 1.965	5.92	F = 0.561	3.9537	F = 1.062	3.4231	F = 1.596	1.9722	F = 2.235	1.5981	F = 0.828	χ^2^ = 2.930
Black	0.9667	*p* = 0.137	5.67	*p* = 0.643	5.4214	*p* = 0.371	4.8571	*p* = 0.198	3.2857	*p* = 0.091	2.7286	*p* = 0.483	*p* = 0.403
White	3.0000		6.33		3.6500		3.2050		3.1000		1.7000		
Mixed	3.0000		7.00		4.7000		4.2000		3.7000		1.4000		
Relationship status													
Married/Living with partner	4.5000	F = 1.639	6.15	F = 0.399	4.2000	F = 0.393	3.2775	F = 0.587	2.0750	F = 0.315	1.4250	F = 0.304	χ^2^ = 3.362
Living alone	4.2159	*p* = 0.208	5.81	*p* = 0.673	3.8962	*p* = 0.677	3.5769	*p* = 0.599	2.4231	*p* = 0.731	1.8231	*p* = 0.739	*p* = 0.186
Living with friends /relatives/supported accommodation	2.4563		6.34		4.5000		3.9594		2.6250		1.6750		
Employment													
Not employed	3.8069	*t* = −1.458	6.06	*t* = 0.290	4.1265	*t* = −1.056	3.6931	*t* = −0.804	2.2157	*t* = −1.945	1.6843	F = 0.370	χ^2^ = 0.044
Employed	6.5000	*p* = 0.156	5.75	*p* = 0.773	5.1429	*p* = 0.296	4.2929	*p* = 0.425	3.9286	*p* = 0.057	1.4286	*p* = 0.713	*p* = 1.000
Education level													
Below A level	4.0750	*t* = 0.260	5.97	*t* = −0.502	4.2794	*t* = 0.358	3.7941	*t* = 0.848	2.3676	*t* = 0.135	1.3824	*t* = −1.206	χ^2^ = 2.410
A level or above	3.8447	*p* = 0.796	6.23	*p* = 0.617	4.0863	*p* = 0.722	3.4225	*p* = 0.399	2.3000	*p* = 0.893	1.8975	*p* = 0.232	p = 0.159
SMI diagnosis													
Schizophrenia	3.5909	F = 0.767	6.07	F = 0.378	4.3182	F = 0.391	3.9348	F = 1.264	2.8261	F = 1.260	2.2273	F = 1.748	χ^2^ = 6.777
Schizoaffective disorder	3.6429	*p* = 0.520	6.10	*p* = 0.823	3.9500	*p* = 0.815	3.9500	*p* = 0.292	2.9000	*p* = 0.294	1.4500	*p* = 0.149	*p* = 0.148
Bipolar disorder	5.3333		6.86		4.7778		4.0556		2.5000		2.4444		
Depression + psychotic feature	3.7000		5.88		3.7308		2.9231		1.6346		1.1731		
More than one diagnosis	–		5.92		4.0556		4.0000		2.5000		1.3889		
Diabetes management													
Tablets	3.3111	F = 2.128	6.07	F = 0.212	4.2779	F = 1.836	3.8433	F = 2.547	2.6442	F = 1.450	**2.0846**	**F = 3.120**	χ^2^ = 4.679
Insulin	5.8750	*p* = 0.114	6.50	*p* = 0.810	4.8750	p = 0.148	4.5000	*p* = 0.062	2.1250	*p* = 0.235	**0.8750**	***p*** **= 0.031**	*p* = 0.197
Tablets and lifestyle	5.8750		5.67		2.0833		2.2500		0.9167		**1.4167**		
Lifestyle only	3.3000		–		4.0000		2.9033		2.0000		**0.6000**

**Table 7:** Multiple linear regressions

		Variable Coefficients			Model Summary
SDSCA Outcome variable	Independent variables	B	Std. Error	p	Cumulative adjusted R^2^
Blood glucose testing	CORE-10 Total	.189	.049	<.001	0.37
	Behavioural regulation	−.740	.188	<.001	
Diet General	Beliefs about Capabilities	.494	.126	<.001	0.42
	Beliefs about Consequences	.363	.156	.020	
	Goals	.745	.198	<.001	
Diet Specific	Behavioural Regulation	−.245	.098	.012	0.19
	Goals	.500	.171	.003	
Exercise	Environmental Context & Resources	.328	.132	.013	0.15
	Goals	.481	.199	.016	
Foot Care	Diabetes mgt – tablets	1.360	.445	.002	0.15
	Emotion	−.307	.120	.011

**Table 8:** Logistic regression

SDSCA Outcome	Independent variables	B	Std. Error	Wald χ2	p	Nagelkerke R^2^
Smoking	Emotion	−.367	.157	5.431	.020	0.10

### Medication taking

Frequency of medication taking was higher in participants who received more of the diabetes healthcare essentials. Associations between medication taking and all other IVs were not statistically significant in univariate analyses therefore multiple regression analysis was not performed.

### Blood glucose testing

More frequent blood glucose testing was associated with psychological distress and *Behavioural Regulation* in the univariate analysis. Both variables remained significant in the multiple regression analysis and explained 37% of the variance in blood glucose testing. More frequent testing was reported by those who scored higher on psychological distress and those who found it less difficult to establish a routine to manage their diabetes.

### General diet

In univariate analysis, following a healthy diet was more frequent in participants who received a higher number of the diabetes healthcare essentials and who reported less psychological distress. All of the TDF domains, with the exception of *Skills* and *Social/Professional Role and Identity*, were also associated with general diet. In the multiple regression analysis, 42% of the variance in general diet was explained by the domains *Beliefs about Consequences, Goals* and *Beliefs about Capabilities*. Eating a healthy diet was more frequent among those who expressed a stronger belief that failure to manage their diabetes would damage their health, gave diabetes a higher goal priority and had greater confidence in their ability to manage their diabetes.

### Specific diet

Seven of the TDF domains - *Behavioural Regulation, Beliefs about Capabilities, Environmental Context and Resources, Goals, Optimism, Reinforcement* and *Social influences* - were associated with frequency of specific healthy diet in the univariate analysis. In multiple regression, 19% of the variance in specific diet was explained by the domains *Goals* and *Behavioural Regulation.* Eating a healthy diet was more frequent among those who gave diabetes a higher goal priority and who reported less difficulty in establishing a routine to manage their diabetes.

### Exercise

In univariate analysis, exercise was more frequent among participants who received a higher number of the diabetes healthcare essentials and who reported less psychological distress. Six of the TDF domains - *Beliefs about Capabilities, Environmental Context and Resources, Goals, Intentions, Reinforcement* and *Social Influences - w*ere also associated with frequency of exercise. In multiple regression, *Goals* and *Environmental Context and Resources* remained significant, explaining 15% of the variance. Exercise was more frequent among those who gave diabetes a higher goal priority and who reported greater access to relevant services for their diabetes.

### Foot care

In univariate analysis, more frequent foot care was reported by those who scored lower on diabetes-related *Emotion* and those whose diabetes was managed with tablets rather than lifestyle only. Both variables remained significant in the multiple regression analysis, explaining 15% of the variance.

### Smoking

Smoking was more prevalent in men than women and in those who scored lower on diabetes-related *Emotion*. Only the domain *Emotion* remained statistically significant in the logistic regression analysis, with smokers scoring lower on diabetes-related emotion.

## Discussion

To the authors’ knowledge, this is the first survey that has asked people with SMI about the factors that affect their ability to manage their diabetes. We found that several aspects of diabetes healthcare and self-management are suboptimal in people with diabetes and SMI and some, though not all, are poorer than in the general diabetes population. Several factors emerged as important for diabetes self-management, including the degree to which participants were receiving recommended diabetes healthcare, the support they received, their emotional wellbeing, the priority they give to diabetes, their perceived ability to manage diabetes or establish a routine to do so and the perceived consequences of not managing their diabetes.

There was variability in the average number of days on which the different diabetes self-management behaviours were performed. The most commonly reported behaviour was taking medication, with most participants reporting taking their diabetes medication every day. However, foot care was infrequent and exercise was taken on average 2 days per week. Participants reported following a healthy diet for approximately half of the week. When compared to both UK and international data from the DAWN2 study in the general diabetes population [9], participants in the current study took their medication and checked their blood sugar at about the same frequency as those in the DAWN2 study, but eating a healthy diet, taking exercise and checking feet were less frequent in the current sample. The rate of smoking in our sample was almost three times the rate in the general population [24], but is similar to smoking prevalence among people with SMI in the UK [25]. Researchers in the DAWN2 study concluded that diabetes self-management is sub-optimal in the general population with diabetes [9] and our findings indicate that people with SMI experience even greater difficulty in self-managing some aspects of their diabetes.

Participants reported that they found taking regular exercise and following a healthy diet particularly difficult. A recent systematic review [26] identified only one small trial (*n* = 64) of a lifestyle intervention for people with diabetes and SMI [27, 28]. The trial reported a small improvement in physical activity immediately following the intervention, which was not maintained at 6-month follow-up. Self-reported calorie intake did not change as a result of the intervention however, improvement was reported in body mass index. The reviewers concluded [26] that there is insufficient evidence to show whether diabetes self-management interventions are effective for people with SMI and further trials of theoretical and evidence-based interventions are needed.

Although the smoking rate was very high in the current study, only 20% of smokers considered that not smoking was the most difficult aspect of managing their diabetes. It may be that people with SMI do not associate smoking with diabetes self-management even though it increases the risk of complications [29]. Over 60% of smokers in our sample reported that they had been given support and advice on how to quit but unfortunately this was clearly ineffective. A recent systematic review of smoking cessation in severe mental illness concluded that specialised smoking cessation programmes did not show evidence of benefit, but effective pharmacological interventions are available [30]. It is crucial that diabetes self-management interventions for people with SMI include appropriate support to give up smoking to help reduce the risk of diabetes complications.

We examined whether people with SMI received recommended diabetes healthcare and whether their diabetes self-management was related to the level of care received. In a replication of the survey conducted by Diabetes UK [18], we found that the percentages reporting receipt of diabetes healthcare essentials were lower in the current sample for some, but not all, aspects of care. Percentages were lower for checks of HbA1c, BP, cholesterol, eyes, feet and kidney function and for developing a care plan with their healthcare professional. Percentages were similar for weight checks and referral to diabetes specialists. The percentage of participants in the current study being offered diabetes education, given advice to quit smoking and offered specialist psychological support was higher than in the Diabetes UK survey. Participants who reported receiving fewer of the healthcare essentials also reported less frequent performance of three diabetes self-management behaviours: taking medication, eating a healthy diet and exercise. Several participants were unaware of whether or not they had received some of the health checks, perhaps suggesting that they are not fully engaged in their diabetes care. In previous research in the UK, two comparisons between diabetes care for those with and without SMI have been conducted in primary care [31, 32]. Whyte et al. [32] examined 17 quality indicators and reported that having SMI did not result in poorer diabetes care. The only significant difference was on target HbA_1c_ level which was better in those with SMI, however the proportions achieving target HbA_1c_ levels were relatively low in both populations (54% in those with SMI v 47% in those without SMI). Mathur et al. [31] found no significant differences in statin prescribing or cholesterol control between people with or without SMI. They found that people with SMI had better HbA_1c_ and blood pressure control than those without SMI but less than half in both populations were within the target range for HbA_1c_. However, those with SMI were more likely than people without SMI to be smokers and to be obese and were less likely to have had retinopathy screening [31]. Our findings on smoking, diet and exercise are consistent with Mathur et al’s [31] finding of higher rates of smoking and obesity. Whyte et al. [32] and Mather et al. [31] did not ask about several aspects of diabetes care, including foot checks, referral to structured education, care plans, psychological support or being seen by a diabetes specialist if admitted to hospital. The percentages of participants in the current study who reported receipt of these aspects of care ranged from 20% being seen by a diabetes specialist in hospital to 65% being offered an education course. It is encouraging that 23% of participants reported that their diabetes care had improved over the previous 12 months, perhaps reflecting the greater emphasis now being placed on the physical health of people with SMI [33]. However, these findings indicate that several aspects of diabetes care remain suboptimal for people with SMI. The finding that survey participants who received more complete diabetes care were significantly more likely to be engaging in self-management activities, such as exercise and healthy eating, indicates the potentially motivating aspects of service contact. It has been suggested that factors such as the high number of contacts people with SMI have with health professionals may confer a benefit for their diabetes medication adherence [34], however the cross-sectional nature of this research means that we cannot infer causation.

The current study identified a number of other important factors that were related to performance of diabetes self-management behaviours. When these factors were examined in multiple regression analyses, the domains of *Goals*, *Behavioural regulation* and *Emotion* or psychological distress were statistically significant across more than one behaviour. The findings suggest that setting diabetes-related goals and action plans, including how to manage diabetes in the face of fluctuations in mental health, may be important for optimising diabetes self-management, supporting our previous qualitative work [11]. Few participants (28.6%), however, had developed a diabetes care plan with their healthcare professional. Emotional factors were also important, but only 40% of participants reported that they had received emotional or psychological support from a specialist healthcare professional or service, which is of particular concern given that all participants had a SMI and over half reported at least moderate psychological distress. A recent study of community mental health care planning found that few service users felt they were adequately involved in developing meaningful care plans [35]. The current study indicates that this is the case for their physical as well as their mental health and only a minority of service users feel that their mental health teams support their diabetes care. Community mental healthcare pathways will therefore need to be radically revised in order to improve physical health outcomes in those with SMI.

Identification of the theoretical domains that appear to be important for diabetes self-management in people with SMI is a step towards the development of an intervention to support this population. Recent expert consensus work [29, 30] has produced a method for mapping theoretical domains onto appropriate behaviour change techniques (BCTs) [31]. For example, *Goal-setting, Review of [outcome and behaviour] goals* and *Action planning* are BCTs suggested to bring about a change in behaviour by altering a person’s *Goals* related to that behaviour [36]. One example of the successful use of action planning in mental health is the Wellness Recovery Action Plan (WRAP) [37] which has been widely-used to help people develop plans for maintaining their health when well and action plans to help manage if they become unwell. Use of WRAPs was found to improve psychiatric symptoms and quality of life [37] and reduce the need for and use of mental health services [38]. WRAPs can be seen to incorporate several of the BCTs listed and could potentially be adapted to include diabetes self-management as well as offering a format for developing more meaningful care plans for people with SMI.

Self-management education has been shown to improve outcomes for the general diabetes population [39–41] and it is encouraging that participants in this study were as likely as the general population with diabetes (perhaps somewhat more likely) to be offered an education course. However this still equated to less than two-thirds being offered an education course and only 60% of participants felt that the available diabetes education met their needs. The recent Cochrane review [26] identified only one education course that had been developed specifically for people with diabetes and SMI [27, 28], and evaluations of other diabetes education programmes often exclude people with SMI [42–44]. Given the challenges that people with SMI face when attempting to manage their diabetes, it is important that appropriate education and support is developed and provided on an ongoing basis. Opportunities for provision of diabetes education and skills development should not be limited to diabetes specialist services but also optimised in primary care and mental health services. The current study has identified several factors associated with diabetes self-management behaviour in people with SMI and suggested some strategies that may help to address these. Development and testing of interventions that target these factors may generate more effective diabetes self-management education programmes for this population.

The study had a number of limitations. The use of online recruitment methods meant that we are unable to estimate response rate, which is a limitation of all surveys that recruit in this way. As the study is cross-sectional, we are also unable to infer causation. Although we tried several routes to recruit participants, the sample size achieved was small, which resulted in some of the analyses being underpowered. Similar difficulties in recruitment have been found in other studies in SMI. For example, a telephone survey of mental health service users conducted across NHS sites between 2008 and 2014 obtained response rates ranging from 6 to 11% [45] and the 2016 Community Mental Health Survey achieved a response rate of 28% [46]. We used an anonymous postal and online survey in the hope that providing anonymity would encourage response and we also offered a small donation to relevant charities. However, providing anonymity meant that we could not send reminders to non-responders, a strategy that has been found to increase response rates in survey research [47], and which was used in the Community Mental Health Survey [46]. Anonymity also precluded us from making payments direct to participants, which may also have improved recruitment.

Given the small sample size, univariate screening was used to identify potentially relevant predictors for the regression models. Although this method has limitations [48], we chose a widely adopted approach that should be seen as hypothesis generating, suggesting avenues for future research. Although this study measured several self-management behaviours, we did not adjust the *p*-value to account for multiple outcomes as it would have increased the likelihood of type II error which is no less important than a type I error. We took this approach as the study is exploratory, but we acknowledge that we may have identified some chance findings.

To avoid the questionnaire being onerously long, we included only one measure of mental health - a short measure of psychological distress [16]. This meant that other potentially important barriers to effective diabetes self-management, such as positive and negative symptoms of psychosis, were not assessed.

Although the education level of participants is similar to that of the general UK population [49], this may not be representative of people with SMI, in whom lower levels of educational attainment have been reported [50]. It is possible that we recruited a sample of participants who are particularly interested in managing their diabetes and that this is an area of relatively low priority for many people with diabetes and SMI. Furthermore, those least likely to be able to manage their diabetes are probably also unlikely to complete a survey. Giving greater priority to physical health, including diabetes, is an important area to be promoted among people with SMI and those involved in their care.

## Conclusions

This study demonstrates that people with SMI find it difficult to manage aspects of their diabetes and many do not receive all of the recommended diabetes healthcare essentials. The ability of respondents to manage their diabetes was influenced by the level of diabetes healthcare and support they received, their emotional wellbeing, the priority they give to diabetes, their perceived ability to manage diabetes or establish a routine to do so and the perceived consequences of their diabetes management. The development and evaluation of tailored interventions that address these areas are needed to help improve diabetes self-management support for people with SMI.

## Additional file

Additional file 1: Barriers and enablers of diabetes self-management questionnaire. Questionnaire items. (DOCX 35 kb)

## Abbreviations

ANOVA: Analysis of variance
BCT: Behaviour change techniques
BP: Blood pressure
EC&R: Environmental Context and Resources
GP: General Practice
HbA_1c_: Glycated haemoglobin (A1c)
IV: Independent variables
MADP: Memory, Attention and Decision Processes
MCAR: Missing Completely at Random
NHS: National Health Service
PPI: Patient and public involvement
SDSCA: Summary of Diabetes Self-Care Activities
SMI: Severe mental illness
SPRI: Social/Professional Role and Identity
TDF: Theoretical Domains Framework
WRAP: Wellness Recovery Action Plan
