# Supplementary material for: Barriers to effective diabetes management – a survey of people with severe mental illness
Source: BMC Psychiatry. 2018 Jun 1;18:165. doi: 10.1186/s12888-018-1744-5 (PMC5984777; doi:10.1186/s12888-018-1744-5)
Supplement: Supplementary file 1 — Barriers and enablers of diabetes self-management questionnaire. Questionnaire items. (DOCX 35 kb) [file 12888_2018_1744_MOESM1_ESM.docx]

**BARRIERS AND ENABLERS OF DIABETES SELF-MANAGEMENT QUESTIONNAIRE.**

**The questions below ask you about how you find managing your diabetes.**

**By ‘managing your diabetes’ we mean:**

- **following a healthy eating plan**
- **exercising for at least 30 minutes, 5 days a week**
- **testing your blood sugar, if you have been asked to**
- **checking your feet**
- **not smoking**
- **taking your medications as recommended**

**There are no right or wrong answers. Please circle the response that relates most closely to your experiences.**

1. **I know about diabetes and its possible complications: [KNOWLEDGE]**

| Strongly disagree | Disagree | Somewhat disagree | Neither agree nor disagree | Somewhat agree | Agree | Strongly agree |
| --- | --- | --- | --- | --- | --- | --- |
| **1** | **2** | **3** | **4** | **5** | **6** | **7** |

1. **I know how to manage my diabetes: [KNOWLEDGE]**

| Strongly disagree | Disagree | Somewhat disagree | Neither agree, nor disagree | Somewhat agree | Agree | Strongly agree |
| --- | --- | --- | --- | --- | --- | --- |
| **1** | **2** | **3** | **4** | **5** | **6** | **7** |

1. **Managing diabetes requires particular skills: [SKILLS]**

| Strongly disagree | Disagree | Somewhat disagree | Neither agree, nor disagree | Somewhat agree | Agree | Strongly agree | |
| --- | --- | --- | --- | --- | --- | --- | --- |
| **1** | **2** | **3** | **4** | **5** | **6** | **7** | |
|  |  |  |  |  |  |  | |
| 1. **Seeing the benefits of managing my diabetes is rewarding: [REINFORCEMENT]**  \| Strongly disagree \| Disagree \| Somewhat disagree \| Neither agree, nor disagree \| Somewhat agree \| Agree \| Strongly agree \| \| --- \| --- \| --- \| --- \| --- \| --- \| --- \| \| **1** \| **2** \| **3** \| **4** \| **5** \| **6** \| **7** \| | | | | | | |  |

1. **I have access to the health services I need to help me manage my diabetes:**

**[ENVIRONMENTAL CONTEXT AND RESOURCES]**

| Strongly disagree | Disagree | Somewhat disagree | Neither agree, nor disagree | Somewhat agree | Agree | Strongly agree |
| --- | --- | --- | --- | --- | --- | --- |
| **1** | **2** | **3** | **4** | **5** | **6** | **7** |
|  |  |  |  |  |  |  |

1. **I find it difficult to control my sugar intake: [BELIEFS ABOUT CAPABILITIES]**

| Strongly disagree | Disagree | Somewhat disagree | Neither agree, nor disagree | Somewhat agree | Agree | Strongly agree |
| --- | --- | --- | --- | --- | --- | --- |
| **1** | **2** | **3** | **4** | **5** | **6** | **7** |

1. **I have access to specialist diabetes services: [ENVIRONMENTAL CONTEXT AND RESOURCES]**

| Strongly disagree | Disagree | Somewhat disagree | Neither agree, nor disagree | Somewhat agree | Agree | Strongly agree |
| --- | --- | --- | --- | --- | --- | --- |
| **1** | **2** | **3** | **4** | **5** | **6** | **7** |
|  |  |  |  |  |  |  |

1. **The available diabetes education meets my needs: [ENVIRONMENTAL CONTEXT AND RESOURCES]**

| Strongly disagree | Disagree | Somewhat disagree | Neither agree, nor disagree | Somewhat agree | Agree | Strongly agree |
| --- | --- | --- | --- | --- | --- | --- |
| **1** | **2** | **3** | **4** | **5** | **6** | **7** |
|  |  |  |  |  |  |  |

1. **If I don’t manage my diabetes properly I will have poor health: [BELIEFS ABOUT CONSEQUENCES]**

| Strongly disagree | Disagree | | Somewhat disagree | Neither agree, nor disagree | Somewhat agree | Agree | Strongly agree |
| --- | --- | --- | --- | --- | --- | --- | --- |
| **1** | **2** | | **3** | **4** | **5** | **6** | **7** |
|  |  | |  |  |  |  |  |
|  | |  |  |  |  |  |  |

1. **If I don’t manage my diabetes properly it will make my mental health worse:**

**[BELIEFS ABOUT CONSEQUENCES]**

| Strongly disagree | Disagree | Somewhat disagree | Neither agree, nor disagree | Somewhat agree | Agree | Strongly agree |
| --- | --- | --- | --- | --- | --- | --- |
| **1** | **2** | **3** | **4** | **5** | **6** | **7** |
|  |  |  |  |  |  |  |
|  |  |  |  |  |  |  |

1. **I am confident that I can manage my diabetes: [BELIEFS ABOUT CAPABILITIES]**

| Strongly disagree | Disagree | Somewhat disagree | Neither agree, nor disagree | Somewhat agree | Agree | Strongly agree |
| --- | --- | --- | --- | --- | --- | --- |
| **1** | **2** | **3** | **4** | **5** | **6** | **7** |
|  |  |  |  |  |  |  |

1. **I find it difficult to establish a routine to manage my diabetes: [BEHAVIOURAL REGULATION]**

| Strongly disagree | Disagree | Somewhat disagree | Neither agree, nor disagree | Somewhat agree | Agree | Strongly agree |
| --- | --- | --- | --- | --- | --- | --- |
| **1** | **2** | **3** | **4** | **5** | **6** | **7** |

1. **Managing my diabetes is confusing: [MEMORY, ATTENTION AND DECISION PROCESSES]**

| Strongly disagree | Disagree | Somewhat disagree | Neither agree, nor disagree | Somewhat agree | Agree | Strongly agree |
| --- | --- | --- | --- | --- | --- | --- |
| **1** | **2** | **3** | **4** | **5** | **6** | **7** |
|  |  |  |  |  |  |  |

1. **Remembering all the things I need to do to manage my diabetes is difficult:**

**[MEMORY, ATTENTION AND DECISION PROCESSES]**

| Strongly disagree | Disagree | Somewhat disagree | Neither agree, nor disagree | Somewhat agree | Agree | Strongly agree |
| --- | --- | --- | --- | --- | --- | --- |
| **1** | **2** | **3** | **4** | **5** | **6** | **7** |

1. **I want to control my diabetes to protect my health: [GOALS]**

| Strongly disagree | Disagree | Somewhat disagree | Neither agree, nor disagree | Somewhat agree | Agree | Strongly agree |
| --- | --- | --- | --- | --- | --- | --- |
| **1** | **2** | **3** | **4** | **5** | **6** | **7** |

1. **I want to control my diabetes so that I can do the things I want to in life: [GOALS]**

| Strongly disagree | Disagree | Somewhat disagree | Neither agree, nor disagree | Somewhat agree | Agree | Strongly agree |
| --- | --- | --- | --- | --- | --- | --- |
| **1** | **2** | **3** | **4** | **5** | **6** | **7** |
|  |  |  |  |  |  |  |

1. **Managing my diabetes is more important than managing my mental health: [GOALS]**

| Strongly disagree | Disagree | Somewhat disagree | Neither agree, nor disagree | Somewhat agree | Agree | Strongly agree |
| --- | --- | --- | --- | --- | --- | --- |
| **1** | **2** | **3** | **4** | **5** | **6** | **7** |
|  |  |  |  |  |  |  |

1. **Managing my mental health is more important than managing my diabetes: [GOALS]**

| Strongly disagree | Disagree | Somewhat disagree | Neither agree, nor disagree | Somewhat agree | Agree | Strongly agree |
| --- | --- | --- | --- | --- | --- | --- |
| **1** | **2** | **3** | **4** | **5** | **6** | **7** |
|  |  |  |  |  |  |  |

1. **I intend to take steps to manage my diabetes in the future: [INTENTION]**

| Strongly disagree | Disagree | Somewhat disagree | Neither agree, nor disagree | Somewhat agree | Agree | Strongly agree |
| --- | --- | --- | --- | --- | --- | --- |
| **1** | **2** | **3** | **4** | **5** | **6** | **7** |

1. **I am optimistic that I will be able to manage my diabetes in the future: [OPTIMISM]**

| Strongly disagree | Disagree | Somewhat disagree | Neither agree, nor disagree | Somewhat agree | Agree | Strongly agree |
| --- | --- | --- | --- | --- | --- | --- |
| **1** | **2** | **3** | **4** | **5** | **6** | **7** |

1. **If my mental health is poor I find it difficult to manage my diabetes: [EMOTION]**

| Strongly disagree | Disagree | Somewhat disagree | Neither agree, nor disagree | Somewhat agree | Agree | Strongly agree |
| --- | --- | --- | --- | --- | --- | --- |
| **1** | **2** | **3** | **4** | **5** | **6** | **7** |
|  |  |  |  |  |  |  |

1. **Managing my diabetes is my responsibility: [SOCIAL/PROFESSIONAL ROLE AND IDENTITY]**

| Strongly disagree | Disagree | Somewhat disagree | Neither agree, nor disagree | Somewhat agree | Agree | Strongly agree |
| --- | --- | --- | --- | --- | --- | --- |
| **1** | **2** | **3** | **4** | **5** | **6** | **7** |

1. **Managing my diabetes worries me: [EMOTION]**

| Strongly disagree | Disagree | Somewhat disagree | Neither agree, nor disagree | Somewhat agree | Agree | Strongly agree |
| --- | --- | --- | --- | --- | --- | --- |
| **1** | **2** | **3** | **4** | **5** | **6** | **7** |

1. **My friends and family help me to manage my diabetes: [SOCIAL INFLUENCES]**

| Strongly disagree | Disagree | Somewhat disagree | Neither agree, nor disagree | Somewhat agree | Agree | Strongly agree |
| --- | --- | --- | --- | --- | --- | --- |
| **1** | **2** | **3** | **4** | **5** | **6** | **7** |

1. **My diabetes specialists help me to manage my diabetes: [SOCIAL INFLUENCES]**

| Strongly disagree | Disagree | Somewhat disagree | Neither agree, nor disagree | Somewhat agree | Agree | Strongly agree |
| --- | --- | --- | --- | --- | --- | --- |
| **1** | **2** | **3** | **4** | **5** | **6** | **7** |
|  |  |  |  |  |  |  |

1. **My GP and practice nurse help me to manage my diabetes: [SOCIAL INFLUENCES]**

| Strongly disagree | Disagree | Somewhat disagree | Neither agree, nor disagree | Somewhat agree | Agree | Strongly agree |
| --- | --- | --- | --- | --- | --- | --- |
| **1** | **2** | **3** | **4** | **5** | **6** | **7** |
|  |  |  |  |  |  |  |

1. **My mental health team help me to manage my diabetes: [SOCIAL INFLUENCES]**

| Strongly disagree | Disagree | Somewhat disagree | Neither agree, nor disagree | Somewhat agree | Agree | Strongly agree |
| --- | --- | --- | --- | --- | --- | --- |
| **1** | **2** | **3** | **4** | **5** | **6** | **7** |
|  |  |  |  |  |  |  |
